# Seasonal Prevalence of Enteropathogenic *Vibrio* and Their Phages in the Riverine Estuarine Ecosystem of South Bengal

**DOI:** 10.1371/journal.pone.0137338

**Published:** 2015-09-04

**Authors:** Subham Mookerjee, Prasenjit Batabyal, Madhumanti Halder Sarkar, Anup Palit

**Affiliations:** Division of Bacteriology, National Institute of Cholera & Enteric Diseases, Indian Council of Medical Research, P- 33, Scheme-XM, CIT Road, Beliaghata, Kolkata, India; Beijing Institute of Microbiology and Epidemiology, CHINA

## Abstract

Diarrheal disease remains an unsolved problem in developing countries. The emergence of new etiological agents (non-cholera *vibrios*) is a major cause of concern for health planners. We attempted to unveil the seasonal dynamics of entero-pathogenic *Vibrios* in Gangetic riverine-estuarine ecosystem. 120 surface water samples were collected for a period of one year from 3 sampling sites on the Hooghly river. Five enteropathogenic *Vibrio* species, *V*. *cholerae* (35%), *V*. *parahaemolyticus* (22.5%), *V*. *mimicus* (19.1%), *V*. *alginolyticus* (15.8%) and *V*. *vulnificus* (11.6%), were present in the water samples. The vibriophages, *V*. *vulnificus ɸ* (17.5%), *V*. *alginolyticus ɸ* (17.5%), *V*. *parahaemolyticus ɸ* (10%), *V*. *cholerae* non-O1/O139 *ɸ* (26.6%) and *V*. *mimicus ɸ* (9.1%), were also detected in these samples. The highest number of *Vibrios* were noted in the monsoon (20–34°C), and to a lesser extent, in the summer (24–36°C) seasons. Samples positive for phages for any of the identified *Vibrio* species were mostly devoid of that particular bacterial organism and *vice versa*. The detection of toxin genes and resistance to β-lactam antibiotics in some environmental enteropathogenic *Vibrio* species in the aquatic niches is a significant outcome. This finding is instrumental in the south Bengal diarrhoeal incidence.

## Introduction

Diarrheal outbreak, a major public health concern in India, is more palpable across the deltaic peninsula of Gangetic West Bengal, a century old diarrhoea endemic zone [1–3]. *Vibrio*s are one of the common etiological agent of diarrheal disease including cholera; gastrointestinal infections, septicaemia etc [4–6]. They are ubiquitous in fresh water, riverine-estuarine environment and have been isolated customarily from coastal zones in most continents [1, 7–9].

Till date majority of the epidemiological and environmental investigations have been focussed towards the understanding of the aetiology of riverine-estuarine *V*. *cholerae* O1, the most common cholera pathogen, along with south Bengal cholera paradigm [10, 11]. But several other species of *Vibrios* including *V*. *cholerae* non-O1/O139, *V*. *vulnificus*, *V*. *alginolyticus*, *V*. *parahaemolyticus*, *V*. *fluvialis* and *V*. *mimicus* are also responsible for diarrhea, gastroenteritis, necrotizing fasciitis, various other skin diseases and septicemia affecting humans worldwide through water contact and consumption [6, 12–14]. Evidences are galore that the rate of infection caused by *Vibrio* spp. has increased over the past few years [15]. *V*. *cholerae* non-O1/O139 are found to be associated with severe traits of gastroenteric infection indicating their role in diarrhoea [3, 16]. *V*. *parahaemolyticus* of specific serotypes are also associated with diarrheal outbreaks in several parts of the world with the earliest cases being reported from Kolkata, India in 1996 [17]. Even *V*. *fluvialis* has also been reported to induce waterborne diarrhoea in deltaic south Bengal [18–20].

In the coastal zone of the Bay of Bengal, seawater intrusion has been a major concern. As the altitude of this region is low, even a small increase in sea-level may have a significant impact on water regime of the coastal area and the delta of West Bengal in the south-eastern part of India. Apart from the study on *V*. *cholerae* O1 and its phages [11], very little has been done to evaluate the ecology of enteropathogenic *Vibrios* and their phages in different but related coastal environments including river and estuaries, despite the perception that this could provide information regarding the early warning and understanding its relation to human vulnerability to the disease [21] in these diarrhoea endemic Gangetic delta of eastern India (West Bengal).

Few earlier works in different aquatic ecosystem hypothesized that different physico-chemical attributes of the aquatic environment affect the abundance, physiological state and pathogenic potential of *Vibrio* spp. [12, 22, 23]. But so far no longitudinal study has ever been undertaken to solve the environmental mystery.

With this basic contextual information, in a yearlong field based study, we attempted to investigate and identify the seasonal abundance pattern and distribution of toxigenic *Vibrios* (with enteropathogenic potentiality) and their phages under changing physico-chemical patterns and environmental drivers in riverine and brackish water environment of south Bengal. This study also aims to ascertain the correlation of environmental factors and enteropathogenic *Vibrios* in understanding their contribution on the diarrheal incidence pattern in the lower Gangetic delta of India.

## Materials and Methods

### Ethical Statement

No specific permissions were required for these locations/activities. The selected sites were accessible to common people and not situated within restricted area, thus no permissions were required to access them. It is further confirmed that the study did not involve any endangered or protected species.

### Sampling

**Fig 1:**
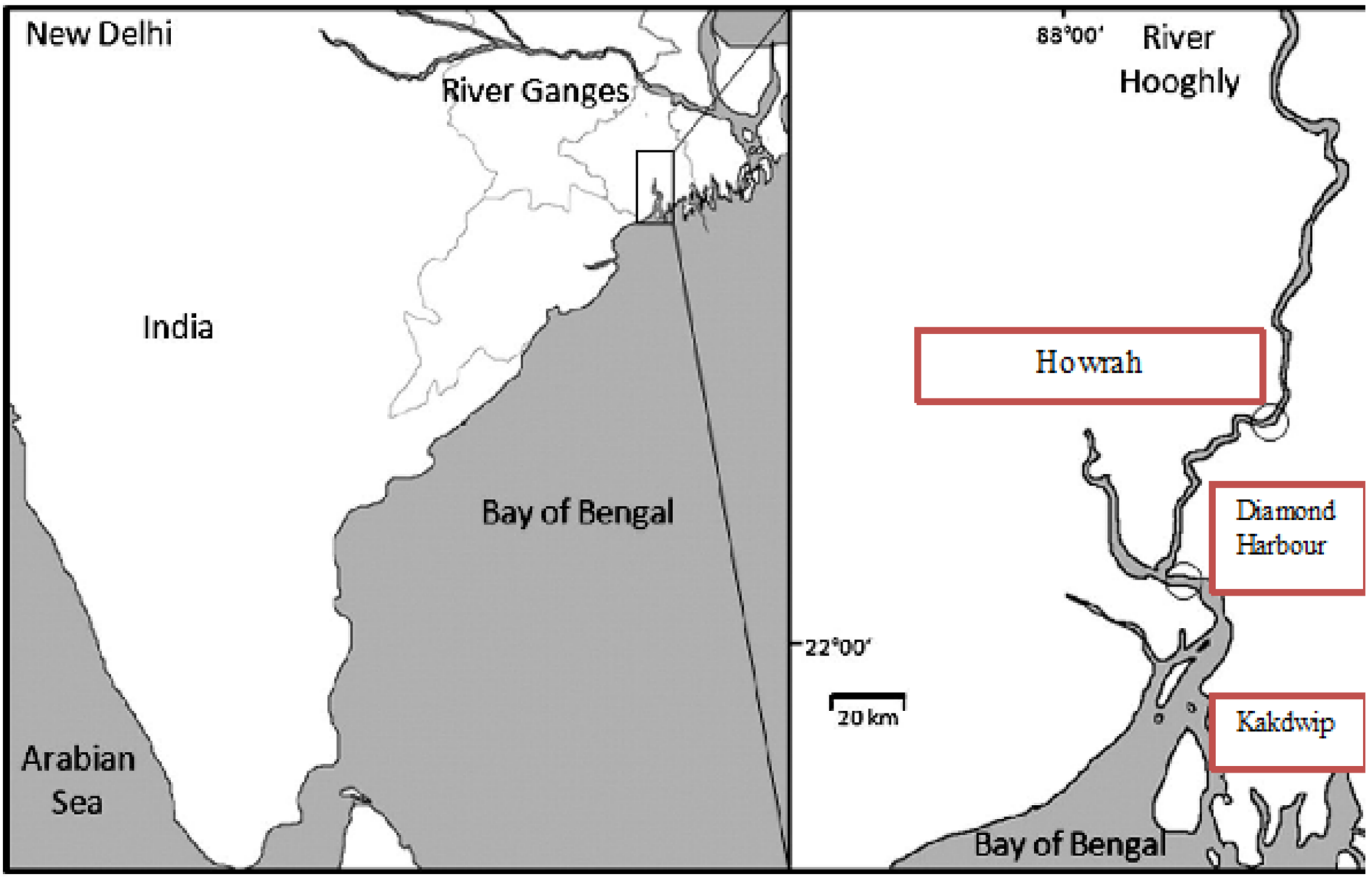
Study area, showing three study points.

During January 2009 to December 2009, a total of 120 water samples were collected from the three sampling sites (40 samples from each site) on the Hooghly River, the main arm of the river Ganges flowing through the southern deltaic West Bengal. The sites were selected based on their respective distances from the sea mouth and navigability (Fig 1).

#### Study sites

Site I. (Howrah; 130km. inland from sea mouth; 22.59°N, 88.31°E):

It lies beside the densely populated city of Kolkata, India. Apart from drinking purposes, water from this area is also accessed for multiple household chores like washing, bathing, cleaning utensils, religious rituals etc. Untreated sewage disposal occurs aplenty from both the banks into the river.

Site II. (Diamond Harbour; 80km. inland from sea mouth; 22.20°N, 88.20°E):

Here river flows through a semi-urban set up. Being in the nearer vicinity to the sea mouth, it is wider with higher aquatic turbulence and receives greater amount of marine brackish water during flood tide. Human intrusion is lesser at this site owing to lower populace, strong water current and greater water exchange with the sea.

Site III. (Kakdwip; 30km. inland from sea mouth; 21.88°N, 88.18°E):

It’s in a rural focus in the district of South 24 Parganas in West Bengal with consistent reports of diarrhoeal endemicity. The river is widest at Site III and records the highest tidal effect among the 3 study sites (Fig 1).

#### Sample Collection procedure

Sampling was carried out every fortnight, in order to embrace the effect of hydrological changes due to alternating high and low tides, as well as during increasing and decreasing moon phases. At each sampling day, 5 samples were collected at regular intervals (every 2 h) covering a 10–12 h period of a tidal cycle including low and high tides. During each sample collection, three to four subsamples of surface water from the midstream were taken with a metal bucket and pooled together in a sterile 10 L container. Subsequently, the samples were shaken and divided into 1 L aliquots.

Immediately after sample collection, temperature, pH, conductivity, turbidity and salinity were measured with a Thermo Orion Multi-meter (pH probe [9157BNMD refillable epoxy pH/ATC triode] and conductivity probe [013605MD K = 0.55] duraprobe). Turbidity was expressed as Nephelometric Turbidity Units (NTU) and salinity was expressed as parts per thousand (ppt). After determination of physico-chemical variants, water samples were transported to the central laboratory in sterile 1L glass bottles at 4°C in dark thermocol boxes.

### Bacteriological Analysis

The samples were processed in accordance with the standard microbiological procedure as described elsewhere [1]. Suspected *Vibrio* colonies were subjected to biochemical confirmation using HIVIBRIO kit (Himedia, India). Biochemically suspected *Vibrio* isolates were further subjected to molecular identification by multiplex PCR (Polymerase Chain Reaction), targeting the *dnaJ* gene for the 5 pathogenic *Vibrio* species viz. *V*. *cholerae*, *V*. *parahaemolyticus*, *V*. *vulnificus*, *V*. *alginolyticus* and *V*. *mimicus* [24].

#### Phenotypic Characterization

PCR confirmed *Vibrio* isolates were further subjected to the salt tolerance test [25]. All the confirmed *Vibrio* isolates of the 5 enteropathogenic species were tested for antibiotic sensitivity pattern by disc diffusion method [26] with commercially available disks (Becton Dickinson, USA).

#### Genotypic Characterization

PCR was performed with purified genomic DNA as template from all the isolated enteropathogenic *Vibrio*s for the detection of the species specific toxin genes associated with pathogenicity of the particular *Vibrio* species. The reaction mixtures consisted of 10X PCR reaction buffer, 2.5mM DNTP mixture, 1.5 mmol l^-1^ MgCl_2,_ 100 nmol l^-1^of primer, 1 U Taq polymerase (Genie, Merck) and 2 μl of template DNA. Nucleotide sequence of the primers and the conditions of the PCR are presented in Table 1.

**Table 1:** List of toxin genes, their primer sequences and PCR conditions.

Gene	Primer Sequence (5’-3’)	Denaturing	Annealing	Extension	Reference
*ctxA*	F-5’CTCAGACGGGATTTGTTAGGCACG 3’	94°C, 90S	60°C, 90S	72°C, 90S	1 (S1 Reference)
	R-5’TCTATCTCTGTAGCCCCTATTACG 3’				
*tcpA* Classical	F-5’CACGATAAGAAAACCGGTCAAGAG 3’	94°C, 90S	60°C, 90S	72°C, 90S	1 (S1 Reference)
	R-5’ACCAAATGCAACGCCGAATGGAGC 3’				
*tcpA* El Tor	F-5’GAAGAAGTTTGTAAAAGAAGAACAC 3’	94°C, 90S	60°C, 90S	72°C, 90S	1 (S1 Reference)
	R-5’GAAAGGACCTTCTTTCACGTTG 3’				
*toxR(V*. *cholerae)*	F-5’CGGGATCCATGTTCGGATTAGGACAC 3’	94°C, 30S	64°C, 30S	72°C, 30S	2 (S1 Reference)
	R-5’CGGGATCCTACTCACACACTTTGATGGC 3’				
*toxT*	F-5’ACTGTCGACGCAAAGCATATTCAGAGA3’	94°C, 40S	55°C, 40S	72°C, 90S	3 (S1 Reference)
	R-5’CGCGGATCCATACAATCGAAAATAGGA 3’				
*RJ*	F-5’TCGTTAGCGTGTCGGTTCGCAGG 3’	94°C, 40S	55°C, 40S	72°C, 90S	4 (S1 Reference)
	R-5’TGCTTTGTACCAGTCACAGATAG 3’				
*LJ*	F-5’GTGAATCTTGATGAGACGCTCTG 3’	94°C, 40S	55°C, 40S	72°C, 60S	4 (S1 Reference)
	R-5’GGTGAGCCAGGCTTATTTGGG 3’				
*zot*	F-5’TCGCTTAACGATGGCGCGTTTT-3’	94°C, 60s	60°C, 60s	72°C, 60s	5 (S1 Reference)
	R-5’AACCCCGTTTCACTTCTACCCA-3’				
*tdh (V*. *parahaemolyticus)*	F-5’GGTACTAAATGGCTGACATC-3’	94°C, 120s	50°C, 120s	72°C, 30s	6 (S1 Reference)
	R-5’CCACTACCACTCTCATATGC-3’				
*toxR (V*. *parahaemolyticus& V*. *alginolyticus)*	F-5’GATTAGGAAGCAACGAAAG 3’	94°C, 60S	54°C, 60S	72°C, 60S	7 (S1 Reference)
	R-5’GCAATCACTTCCACTGGTAAC 3’				
*tlh*	F-5’AGCGGATTATGCAGAAGCAC 3’	94°C, 60S	54°C, 60S	72°C, 60S	7 (S1 Reference)
	R1-5’GCTACTTTCTAGCATTTTCTCTGC 3’				
	R2-5’ATCTCAAGCACTTTCGCACG 3’				
*vvh*	F-5’CTCACTGGGGCAGTGGCT 3’	94°C, 15S	58°C, 15S	72°C, 20S	8 (S1 Reference)
	R-5’CCAGCCGTTAACCGAACCA 3’				
*tdh (V*. *mimicus)*	F-5’GGTACTAAATGGCTGACATC 3’	94°C, 30S	55°C, 30S	72°C, 30S	9 (S1 Reference)
	R-5’CCACTACCACTCTCATATGC 3’				
*vmh*	F-5’GGTAGCCATCAGTCTTATCACG 3’	94°C, 30S	55°C, 30S	72°C, 30S	9 (S1 Reference)
	R-5’ATCGTGTCCCAATACTTCACCG 3’				

### Vibriophage assay

All riverine-estuarine samples were subjected to the identification of five vibriophages of the afore-mentioned five enteropathogenic *Vibrio* species, following a standard protocol [27, 28] with certain modifications. Briefly, each sample was membrane filtered through 0.22μm (Millipore) membrane and 10ml of the filtrate was mixed with 100μl of MgCl_2_.6H_2_O and kept at 37°C for 5 min. Thereafter, the mixture was mixed with 1ml of log phase culture of each of the standard strains of 5 identified *Vibrio* spp. and 10 ml of melted Luria Agar (BD, USA). Subsequently, the mixture, poured in a petridish, was kept at room temperature to solidify. Afterwards, the plates were incubated overnight at 37°C and Vibriophages were identified by the formation of plaques.

### Statistical Analysis

The results have been analysed statistically applying correlation methods to compare the physico-chemical parameters of water samples and presence of *Vibrios*. For comparisons of parameters each sample data were considered. Further correlations probability values < 0.05 were considered significant. The analysis was done by using Epi Info (Ver 3.5.1. USA).

## Results

Water samples collected from the riverine estuarine sources were analysed for different physico-chemical indices as well as for the entero-pathogenic *Vibrios*, their toxicity and for Vibriophages.

### Physico-chemical Analysis

Throughout the study period, water temperature oscillated between 17.3 to 34.7°C at Howrah, between 15.1 to 35.6°C at Diamond Harbour and 13.4 to 35.6°C at Kakdwip, without any significant variation amongst respective sampling sites. pH level varied between 7.10–8.13 at Howrah site, 7.1–7.96 at Diamond Harbour and 7.67–8.26 at Kakdwip respectively with a basic alkaline drift. Salinity level at Site I (Howrah) always remained <0.1ppt and yearlong salinity gradient varied between 0.2–4.7 ppt. at Site II (Diamond Harbour) and 6.6–14.2 ppt. at Site III (Kakdwip) respectively. Turbidity was slightly higher at Diamond Harbour varying between 86 to 723 NTU, compared to Howrah (32 to 589 NTU) and Kakdwip (6 to 103 NTU).

### Enteric *Vibrios*

*V*. *parahaemolyticus* could be isolated from 27/120 (22.5%) samples, being mostly prevalent in the high saline zone (Site III) (21/27), to some lesser extent at Diamond Harbour (6/21) and was completely absent at Howrah (Fig 1). 19 samples (15.8%) were found to harbour *V*. *alginolyticus* and *V*. *vulnificus* could be isolated from 14 (11.6%) samples. Both *V*. *alginolyticus* and *V*. *vulnificus* could be detected from all the three sampling sites, with a slightly higher preponderance at Site III (Fig 1). *V*. *mimicus* was isolated from 23 water samples (19.1%) with highest preponderance at Site I (12/23), followed by Site II (8/23) and Site III (3/23) respectively (Fig 1). *V*. *cholerae* non-O1/O139 was the most prevalent enteropathogen among all the five species of non-cholera *Vibrios*, which was present in 42 (35%) water samples. Highest abundance of *V*. *cholerae* non-O1/O139 was also observed at Site I (24/42) at salinity <0.1ppt. *V*. *cholerae* O1 was present in 19 (15.8%) water samples and showed distinct seasonality as reported in our earlier studies [11]. Seasonal prevalence of *Vibrios* was highest in the monsoon months (20–34°C), followed by summer (24–36°C) and winter (13–18°C) months respectively. Irrespective of any organism or any site, monsoon seems to be the most favourable condition with maximum isolation rate achieved during the period.

### Salt Tolerance test

It was evident from the salt tolerance test that *V*. *parahaemolyticus* had higher survival rate at higher (2–10%) salt concentration, whereas *V*. *cholerae* non-O1/O139 showed their sustenance at < 1.5% salt concentrations. *V*. *alginolytiocus*, *V*. *vulnificus* and *V*. *mimicus* showed maximum viability at **<**3% salt concentrations.

### Toxin genes

The toxin gene identification of the 5 enteropathogenic *Vibrio* species revealed a lesser degree of existence of toxin genes in the environmental isolates (Table 2). While, presence of accessory cholera toxin genes like *tcp*, *Zot*, *toxR* have been detected from riverine-estuarine *V*. *cholerae* isolates, *V*. *parahaemolyticus* harboured *toxR* and *tdh* genes. Simultaneously toxin genes like *vmh* and *tdh* were present in the *V*.*mimicus* isolates. Among *V*. *vulnificus* population, hemolysin gene (*vvh*) was present and thermolabile hemolysin gene (*tlh*) was detected among *V*. *alginolyticus*.

**Table 2:** Detection rate (%) of entero-pathogenic *Vibrio* species with toxin genes.

Organisms	Total no. of isolates	*ctxA*	*tcpA* Classical	*tcpA*El Tor	*ToxR*	*toxT*	*RJ* &*LJ*	*Zot*	*tdh*	*tlh*	*vvh*	*vmh*
*V*. *cholerae*	42	0	0	4.7%	23.8%	2.3%	11.9%	16.6%	0	0	0	0
*V*. *parahaemolyticus*	27	0	0	0	22.2%	0	0	0	14.8%	0	0	0
*V*. *alginolyticus*	19	0	0	0	21.0%	0	0	0	0	10.5%	0	0
*V*. *vulnificus*	14	0	0	0	0	0	0	0	0	0	7.1%	0
*V*. *mimicus*	23	0	0	0	0	0	0	0	4.3%	0	0	26.1%

### Vibriophage analysis

**Fig 2:**
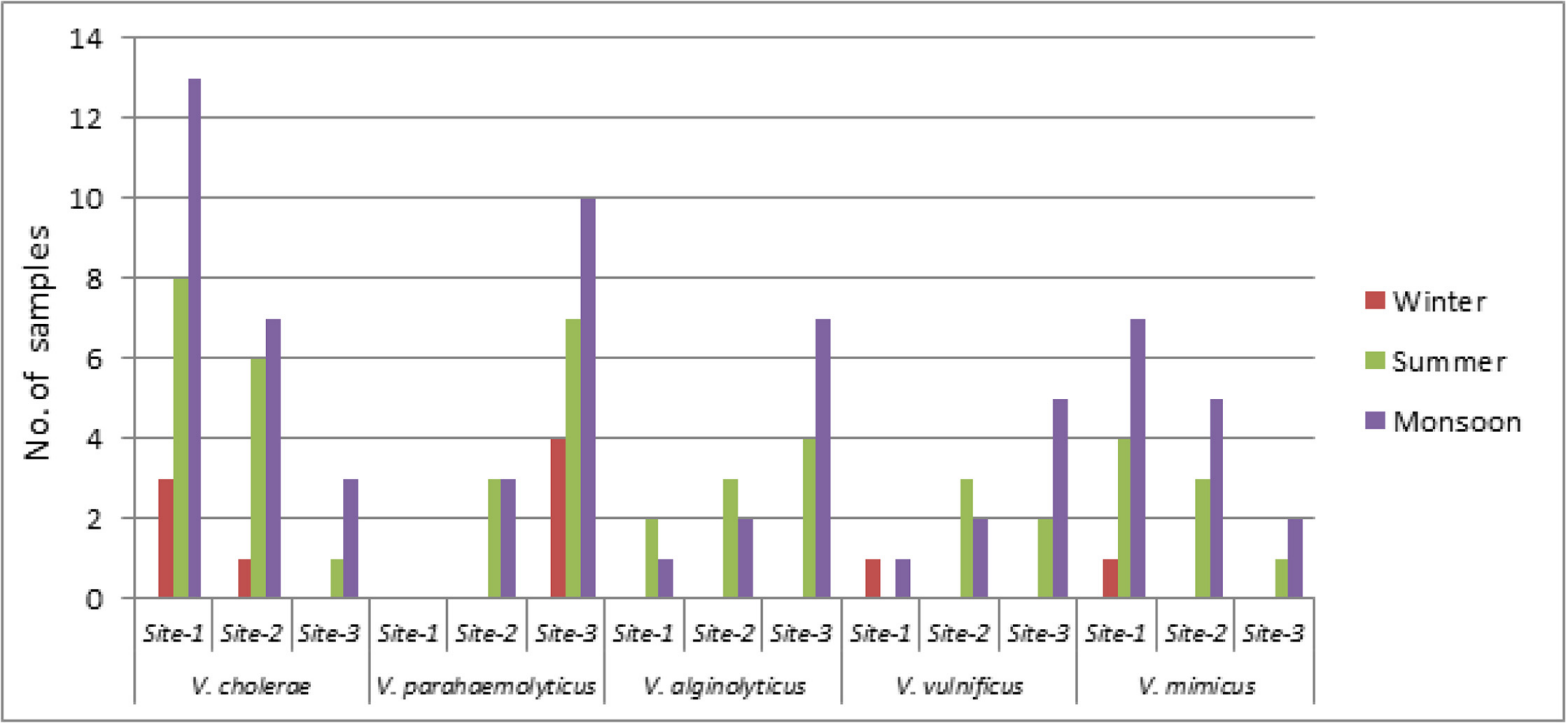
Seasonal abundance of enteric *Vibrio* species at all the sampling sites.

Phages of both *V*. *vulnificus* and *V*. *alginolyticus* could be detected in 21 riverine-estuarine water samples. Higher preponderance of *V*. *vulnificus* phage and *V*. *alginolyticus* phage was observed at Kakdwip (Site III) followed by Diamond Harbour (Site II) and Howrah (Site I). Likewise, *V*. *parahaemolyticus* phage was detected from 12 riverine-estuarine samples collected only from high saline zone of Kakdwip (Site III). On the contrary, *V*. *cholerae* non-O1/O139 phage could be traced in 32 samples collected from Howrah and Diamond Harbour. A lesser persistence of *V*. *cholerae* non-O1/O139 phage has been observed at the high saline regions during monsoon. *V*.*mimicus* phage showed a greater prevalence at Site II and Site I than that of Site III, being present in 11 samples. Irrespective of study sites, higher load of Vibriophages has always been noted in monsoon months followed by summer and winter (Fig 2).

### Antibiotic sensitivity Profile

Irrespective of any species and retention of any toxin genes, more than 80% of the identified enteric *Vibrio* isolates were sensitive to streptomycin, chloramphenicol, cotrimoxazole, tetracycline, fluoroquinolone and cephalosporin, whereas 75% of the *Vibrio* isolates showed resistance to β-lactam derivatives and 40% were resistant to the furazolidone.

## Discussion

A salinity dependant zone demarcation can therefore be drawn from the yearlong evaluation of the salinity gradients of the water samples collected from the three study sites, viz. a “sweet water” or “no saline” zone (Site I; Howrah; <0.1ppt), a “medium saline” zone (Site II; Diamond Harbour; 0.1–5 ppt) and a “high saline” zone (Site III; Kakdwip; 6–15ppt), where the water salinity is inversely proportional to their proximity from the sea mouth. At site II and III, highest salinity was experienced during the summer months, attributable to greater inflow of the sea water. On the contrary lowest salinities were recorded during rainy season due to heavy downpours coupled with mixing of flood water from the adjoining areas, release of water from dams etc.

Water temperature varied uniformly at all the three study sites, with the highest recorded temperature in the summer followed by monsoon months and the lowest temperature in winter. The pH remained at a constant (7.6±0.5) at all the three study sites and showed its basic alkaline nature. A slightly elevated pH at Diamond Harbour can be explained by its higher water turbulence resulting in re-suspension of sediments ensuing in higher turbidity as well as pH. However, highest alkalinity during monsoon months could be attributed to the inflow of the flood water, from the adjoining surroundings, which carry in large amount of organic debris.

Among potentially enteropathogenic *Vibrios*, prevalence of *V*. *parahaemolyticus* in riverine-estuarine environment was directly proportional to the salinity gradient with its highest abundance at Kakdwip (21/27) (Site III; high saline zone) decreasing gradually along with the decrease in salinity being almost undetectable at Howrah. Irrespective of any seasonal fluctuation, consistently higher preponderance of *V*. *parahaemolyticus* at high saline zone indicates their halophilic environmental preference. Statistically, highly significant association between salinity and *V*. *parahaemolyticus* (Table 3) can be attributed to its salt tolerance capacity (2–10% salt concentration) as also evident from the salt tolerance test. Simultaneously, their presence at mid saline zone (Diamond Harbour) during summer and monsoon demonstrate their osmo-regulation capacity as well as a seasonal inland invasion along with marine saline water. *V*.*parahaemolyticus* showed positive correlation with temperature and tide (Table 3). *V*. *parahaemolyticus* phage could only be detected from 10% (12/120) of the samples, all of which were collected from Site III viz. Kakdwip (high saline zone). Presence of *V*. *parahaemolyticus* phage in high saline environment again indicates the preference of saline region as a suitable niche.

**Table 3:** Statistical correlation between physico-chemical variants and abundance of different enetropathogenic *Vibrio* species.

	TEMP (^O^C)	SAL (PSU)	TURB (NTU)	TIDE	*V*.*cholerae*	*V*. *parahaemolyticus*	*V*. *alginolyticus*	*V*. *mimicus*	*V*. *vulnificus*
TEMP (^O^C)	X	+	NS	NS	++	++	++	++	++
SAL (PSU)		X	+	++	-	++	+	-	+
TURB (NTU)			X	NS	++	-	+	++	+
TIDE				X	+	++	+	+	+
*V*.*cholerae*					X	-	NS	++	NS
*V*. *parahaemolyticus*						X	NS	-	NS
*V*. *alginolyticus*							X	NS	+
*V*. *mimicus*								X	+
*V*. *vulnificus*									X

**++** indicate (P < 0.001) highly significant positive correlation, **+** indicate (p < 0.05) significant positive correlation, **NS** indicate (p > 0.05) not significant, - indicate (p < 0.05) significant negative correlation.

**Fig 3:**
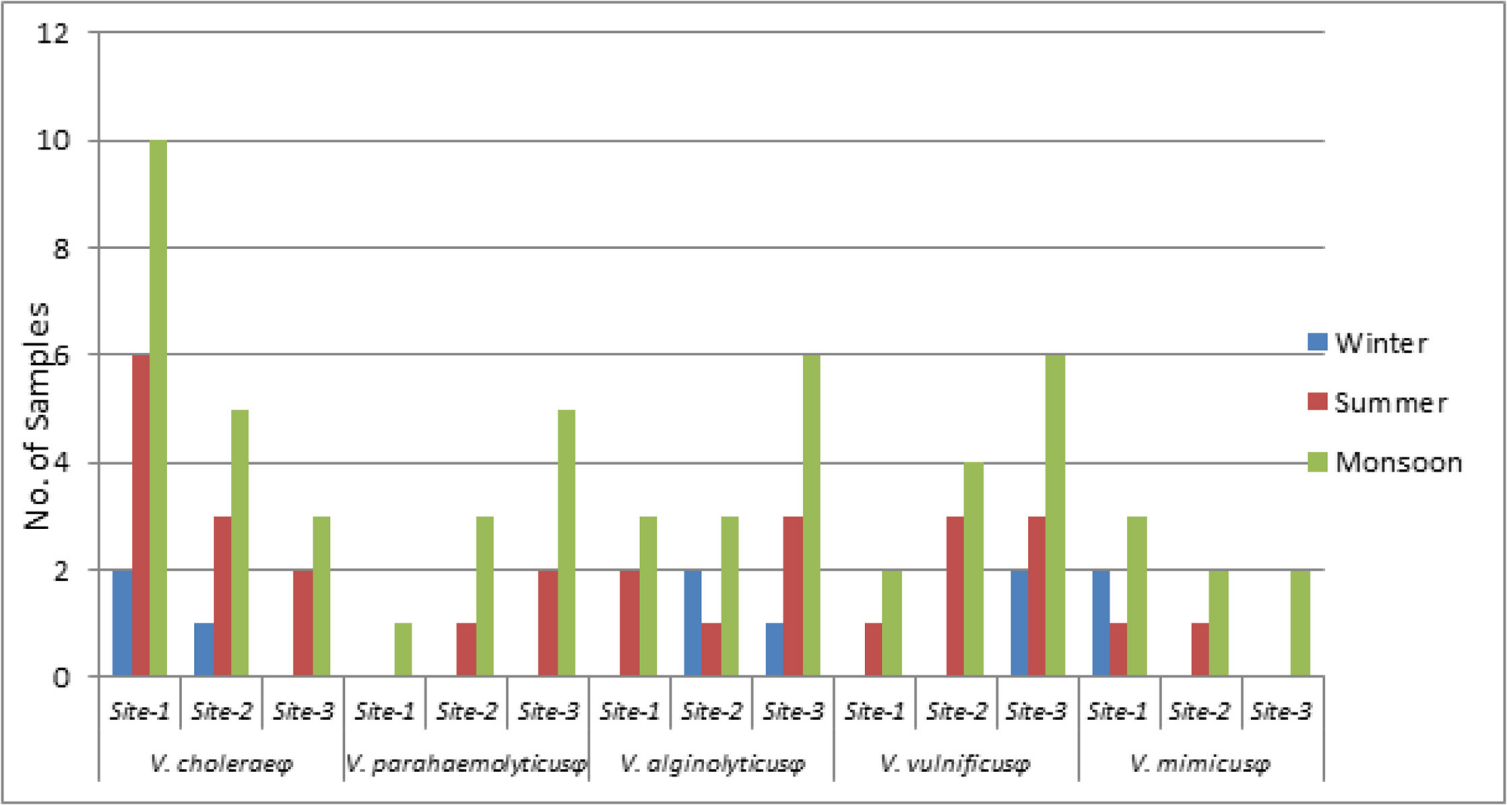
Seasonal abundance of species specific Vibriophages at all the sampling sites.

Similarly, *V*. *alginolyticus* (15.8% samples) and *V*. *vulnificus* (11.6% samples) distribution pattern also reflected higher preponderance at higher salinity zone, with significant association with salinity, despite its presence in other two sites as well. This pattern is possibly attributable to their greater salt tolerance (up to 3%) capacity coupled with osmo-regularity. *V*. *vulnificus* and *V*. *alginolyticus* also showed their positive correlation with temperature, turbidity and tide (Table 3). *V*. *vulnificus* phage and *V*. *alginolyticus* phage was present in higher number of samples, 17.5% each, more than that of *V*. *parahaemolyticus* (Fig 3). Higher abundance of their phages has been observed at high saline region. Coexistence of *V*. *alginolyticus*, *V*. *vulnificus* and *V*. *parahaemolyticus* in estuarine environment indicates their suitability in high saline condition, whereas, increasing number of diarrhoeal cases by these bacterial agents in inland Gangetic delta vindicate their transmission capability and adaptability in the sweet water environment.

Dispositional variation of *V*. *cholerae* (35% of the samples) and *V*. *mimicus* (19.1% samples) was inversely proportional with the riverine salinity gradient (increase in salinity decreases their preponderance). Significant association with turbidity has been noticed for *V*. *cholerae* and *V*. *mimicus* abundance (Table 3). Presence of *V*. *cholerae* in low saline region in Hooghly river is another evidential proof of environmental stimulus in south Bengal cholera menace [11]. *V*. *cholerae* non-O1/O139 phages (26.6% samples) were present in samples collected from all the sampling sites. Higher preponderance of *V*. *cholerae* non-O1/O139 phage was detected from Howrah followed by Diamond Harbour and Kakdwip respectively. *V*. *mimicus* phage was present in 9.1% samples isolated mostly from Howrah, a phenomenon, which reflects their prevalence in low saline zone. Therefore, dynamics of *V*. *cholerae* and *V*. *mimicus* at inland sites of Hooghly river establish the critical role of Hooghly riverine-estuarine ecosystem in south Bengal diarrheal incidence.

**Fig 4:**
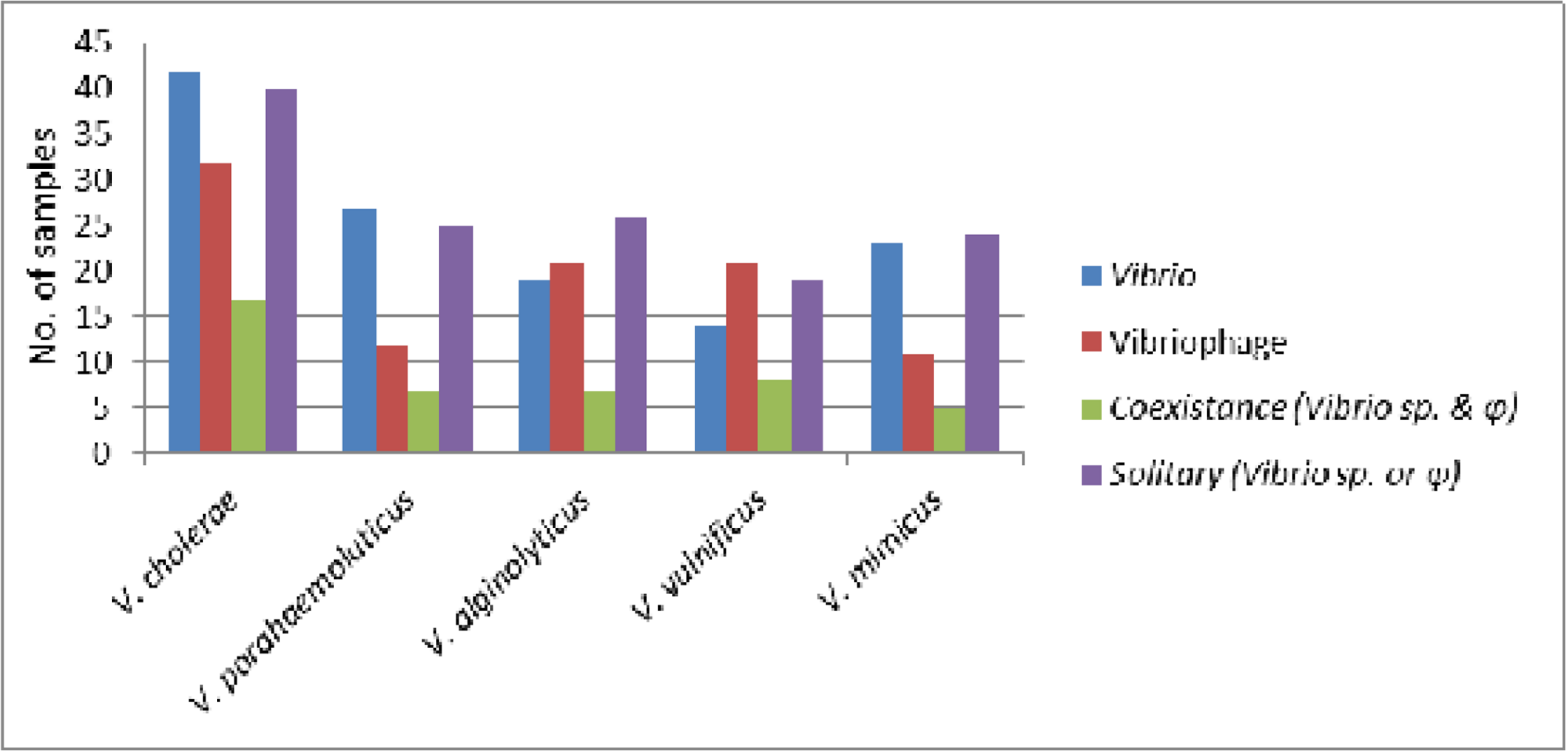
The species specific abundance of *Vibrio*, Vibriophages, their coexistence and solitary existence at all the sampling sites.

Abundance of vibriophages in the riverine estuarine environment indicates a strong seasonal association, where it has been observed that all the phages showed their highest availability during monsoon period irrespective of any sites. Thus it can be hypothesised that alkaline pH along with higher load of organic debris enhances the propagation of the vibriophages during monsoon. At all the sites, solitary existence of either the vibriophages or their respective hosts (Vibrios) has been noticed, which indicate the dominance of any one of the entity (either vibrio or vibriophages) (Fig 4), similar to our previous observations for *V*. *cholerae* O1 [11]. Therefore it can be concluded that phages can be a potential delimiting factor for its host in the aquatic environment.

Ecological conditions of riverine-estuarine environment and abundance of enteric *Vibrios* established a distinct relation between different abiotic factors like water temperature and salinity with the presence of enteric *Vibrios* and its phages (Table 3).

From seasonal distribution pattern of enteropathogenic *Vibrios*, monsoon seems to be the most conducive for their preponderance in riverine-estuarine environment. Although heavy rainfall alters the physico-chemical indices in the aquatic milieu, subsequent intrusion of flood water along with organic debris facilitate the growth of the potential enteropathogenic *Vibrios*.

Among the pool of environmental enteric bacteria, isolated from estuarine region, this is the first study which reports the retention of toxin genes among them. However, toxin genes, that could be found among the environmental isolates were neither in complete cassettes (absence of *ctx* or *tcp* gene), nor in high frequency as also observed in clinical studies from the same focus [16]. These observations support our earlier hypothesis of genetic modification of *Vibrios* through acquisition of genes in aquatic environment [29, 30]. Identification of toxin genes among environmental enteric *Vibrios* is a very important outcome which can explain the emergence of non cholera *Vibrios* as aetiological agents of diarrhoeal menace. Detection of *tcpA* El Tor gene which encodes the pilus colonization factor in two (2) *ctxA* negative environmental *V*. *cholerae* isolates, along with *toxT* gene indicate their colonization potential in gut environment [31]. Previous reports corroborates the prevalence of *rtxA*, *hlyA* and *zot* genes in the environmental *V*. *cholerae* isolates [31], and these genes can also impart virulence to these *V*. *cholerae* isolates.

The *tdh* and *toxR* genes could also be detected in the *V*. *parahaemolyticus* isolates of aquatic origin indicating their virulence potential and capability of causing gastroenteritis. The presence of the virulence genes as *toxR* and *tlh* in *V*. *alginolyticus*, *tdh* and *vmh* in *V*. *mimicus* suggests that other non-cholera *Vibrio* species like *V*. *alginolyticus* and *V*. *mimicus* may also be important reservoirs of many known virulence genes in the aquatic environment. The presence of the *vvhA* gene which encodes a cytotoxin hemolysin protein in *V*. *vulnificus* isolate of aquatic origin is a unique natural phenomenon reported herewith for the first time.

The presence of these toxin genes in the *Vibrios* isolated from the aquatic environment indicates the presence of these genes in the aquatic niches and the possible role of the physico-chemical and environmental attributes in the horizontal gene transfer which appears to take place in the Gangetic riverine estuarine ecosystem as a regular phenomenon, as has been reported earlier in *V*. *cholerae* [30].

The observed *Vibrio* dynamics can very significantly be extrapolated on the diarrheal incidence pattern in southern districts of West Bengal, where from a steady report of higher numbers of diarrheal incidences are recorded every year, showing a parallel incidence peak in monsoon followed by summer months [1, 17, 32]. Abundance of *Vibrios* in the monsoon months may therefore be conclusively attributed to conducive environmental and hydrological indices (temperature, humidity, salinity etc.) coupled with inflow of turbid flood water of increased suspended particulate matter (SPM) into the riverine system brought in by sweeping of organic debris and fecal disposals.

In most developing countries, the treatment of diarrheal diseases by an inadequate quantity of irrationally selected antimicrobials, without any identification/knowledge of causative pathogen is common and is a major contributing factor for multidrug resistances [10]. Since the antibiotic resistant pattern of some enteropathogenic bacteria showed a higher resistance against multiple antibiotics [10], isolation of drug susceptible enteropathogenic *Vibrios* indicates their lack of earlier exposures to different antibiotics in the environmental condition. In spite of showing sensitivity towards most of the conventional antibiotics, the observed resistance to the β-lactam group derivatives highlights that these *Vibrio* community has the potential of acquiring other antibiotic resistant genes enroute inland river water under amiable environmental oscillations [30] or may possibly by usage of the MATE efflux pump to acquire the antibiotic resistance (Batabyal et al, Unpublished data).

In developing countries a large population depends on treated surface waters for drinking, conventional usages and that riverine surface water also contains large number of the different enteropathogenic *Vibrios* with toxin genes and resistance to some antibiotics, resulting in the diarrheal diseases by the fecal–oral contamination [10]. Thus it is of the utmost importance for the common people to avoid direct usage of the natural waters and it is strongly recommended to equip the healthcare system in the third world countries (where diarrhea is a usual entity) for “safe water supply” by under taking regular planned potable water surveillance.

## Conclusion

Thereby it is convincingly established that Gangetic riverine-estuarine aquatic ecosystem regulates the survival, distribution and transmission of diarrheogenic *Vibrios* from its saline habitat to inland fresh water riverine ecosystem, where it can adversely affect human health. Although a flowing aquatic ecosystem like river Ganges harbours different types of bacterial community, detection of entero-pathogenic *Vibrios* along with its phages, the first of its kind, through a yearlong systematic surveillance indicate the role of Gangetic riverine-estuarine ecosystem, on sustained diarrhoeal disease transmission in south Bengal. Further, the retention of toxin genes within *Vibrio* indicates the possibility of aquatic environment induced genetic modification in these *Vibrios* as has been earlier been demonstrated elsewhere [30] in *V*. *cholerae* O1 and may thereby pose a very serious threat of unknown dimension to human health. Current observation of aquatic environmental circulation of enteropathogenic *Vibrios* along the riverine-estuarine gradient also necessitates further long term studies on *Vibrio* dynamics emphasizing their pathogenicity for a comprehensive understanding of the seasonal occurrence of *Vibrio* induced diarrhoea in this endemic focus.

## Supporting Information

S1 Reference References for the toxin genes, their primers & PCR conditions. (DOCX)
