# Supplementary material for: Seasonal Prevalence of Enteropathogenic Vibrio and Their Phages in the Riverine Estuarine Ecosystem of South Bengal
Source: PLoS One. 2015 Sep 4;10(9):e0137338. doi: 10.1371/journal.pone.0137338 (PMC4560433; doi:10.1371/journal.pone.0137338)
Supplement: S1 Reference — (DOCX) [file pone.0137338.s001.docx]

**S1_Reference (for Table-1)**

1. Keasler SP, Hall RH. Detecting and biotyping *Vibrio cholerae* O1 with multiplex polymerase chain reaction. Lancet. 1993; 341: 1661.
2. Ghosh C, Nandy RK, Dasgupta SK, Nair GB, Hall RH, Ghose AC. A search for cholera toxin (CT), toxin coregulated pilus (TCP), the regulatory element ToxR and other virulence factors in non-O1/non-O139 *Vibrio cholerae*. Microb Pathog. 1997; 22: 199-208.
3. Carroll PA, Tashima KT, Rogers MB, DiRita VJ, Calderwood SB. Phase variation in *tcpH* modulates expression of the *ToxR* regulon in *V.cholerae.* Mol Microbiol. 1997; 25(6): 1099–1111.
4. Mitra RK, Nandy RK, Ramamurthy T, Bhattacharya SK, Yamasaki S, Shimada T, et al. Molecular characterization of rough variants of *V.cholerae* isolated from hospitalized patients with diarrhoea. J Med Microbiol. 2001; 50: 268-276.
5. Rivera IN, Chun J, Huq A, Sack RB, Colwell RR. Genotypes associated with virulence in environmental isolates of *Vibrio cholerae*. Appl Environ Microbiol. 2001; 67: 2421-2429.
6. Tada J, Ohashi T, Nishimura N, Shirasaki Y, Ozaki H, [Fukushima S](http://www.ncbi.nlm.nih.gov/pubmed/?term=Fukushima%20S%5BAuthor%5D&cauthor=true&cauthor_uid=1480187), et al. Detection of thermostable direct hemolysin gene (*tdh*) and the thermostable direct hemolysin-related hemolysin gene (*trh*) of *Vibrio parahaemolyticus* by polymerase chain reaction. Mol Cell Probes. 1992; 6: 477–487.
7. Xie ZY, Hu CQ, Chen C, Zhang LP, Ren CH. Investigation of seven *Vibrio* virulence genes among *Vibrio alginolyticus* and *Vibrio parahaemolyticus* strains from the coastal mariculture systems in Guangdong, China. Lett Appl Microbiol. 2005; 41: 202-207.
8. Panicker G, Myers ML, Bej AK. Real-time PCR detection of *Vibrio vulnificus* in oysters: comparison of oligonucleotide primers and probes targeting vvhA. Appl Environ Microbiol. 2005; 71: 5702-5709.
9. Shi L, Miyoshi S, Bi K, Nakamura M, Hiura M, Tomochika K, et al. Presence of Hemolysin Genes (vmh, tdh and hlx) in Isolates of Vibrio mimicus Determined by Polymerase Chain Reaction. J Health Sci. 2000; 46(1): 63-65.
